# Evaluation of the antibacterial effects of four essential oils on antibiotic-resistant bacteria isolated from ventilator-dependent patients

**DOI:** 10.3205/dgkh000620

**Published:** 2026-02-06

**Authors:** Mohammadamin Shabani, Mohammadhassan Tajvidi-Monfared, Zahra Taheri-Kharameh, Faraz Mojab, Saeed Shams, Hassan Vahidi Emami, Iman Khahan-Yazdi

**Affiliations:** 1 Student Research Committee, Qom University of Medical Sciences, Qom, Iran; 2 Student Research Committee, Mazandaran University, Mazandaran, Iran; 3 Spiritual Health Research Center, Department of public Health, School of Health, Qom University of Medical Sciences, Qom, Iran; 4 School of Pharmacy, Pharmaceutical Sciences Research Center, Department of Pharmacognosy, Shahid Beheshti University of Medical Sciences, Tehran, Iran; 5 Cellular and Molecular Research Center, Qom University of Medical Sciences, Qom, Iran; 6 Department of Microbiology and Immunology, Faculty of Veterinary Medicine, University of Tehran, Iran

**Keywords:** Oliveria decumbens, Zataria multiflora, Cuminum cyminum, Trachyspermum ammi, Escherichia coli, Klebsiella pneumoniae, Pseudomonas aeruginosa, antibiotic resistance

## Abstract

**Background:**

Antibacterial resistance has become a critical global health concern. In recent years, significant efforts have been made to discover and utilize natural plant-based products as alternative antibacterial agents. This study aims to evaluate the antibacterial effects of essential oils from four medicinal plants against drug-resistant bacteria isolated from tracheal cultures of ventilator-dependent patients.

**Materials and methods:**

Essential oils were extracted from *Oliveria* *decumbens*, *Zataria* *multiflora*, *Cuminum* *cyminum*, and *Trachyspermum* *ammi* using a Clevenger apparatus. The antibacterial efficacy was tested against drug-resistant bacterial strains, including four strains of *Escherichia (E.) coli*, five strains of *Klebsiella (K.) pneumoniae*, and four strains of *Pseudomonas (P.) aeruginosa*, all isolated from the sputum of ventilator-dependent patients. The disk diffusion method was used to assess antibacterial activity, and the minimum inhibitory concentration (MIC) method was employed to evaluate the antibacterial properties of the two most effective essential oils.

**Results:**

The antibiogram results demonstrated that *Trachyspermum ammi * produced the largest inhibition zones against all bacteria, followed by *Oliveria decumbens*. *Trachyspermum ammi* showed the highest antibacterial activity against *E. coli*, while *Oliveria decumbens* was most effective against *K. pneumoniae*. In MIC testing, Oliveria decumbens exhibited a stronger antibacterial effect at lower concentrations compared to *Trachyspermum ammi*.

**Conclusion:**

This study is the first to report the antibacterial effects of the essential oils from all four plants, particularly *Trachyspermum ammi* and *Oliveria decumbens*, against bacteria isolated from ventilator-dependent patients. Both plants show promising potential as antibacterial agents against these drug-resistant bacteria.

## Zusammenfassung

**Hintergrund:**

Antibiotikaresistenz ist zu einem kritischen globalen Gesundheitsproblem geworden. In den letzten Jahren wurden erhebliche Anstrengungen unternommen, um natürliche pflanzliche Produkte als alternative antibakterielle Mittel aufzufinden und zu nutzen. Die Studie zielt darauf ab, die antibakteriellen Effekte von etherischen Ölen aus vier Heilpflanzen gegen arzneimittelresistente Bakterien zu bewerten, die aus Trachealkulturen von beatmungspflichtigen Patienten isoliert wurden.

**Methode:**

Etherische Öle wurden aus *Oliveria decumbens*, *Zataria multiflora, Cuminum cyminum* und *Trachyspermum ammi* mit einem Clevenger-Apparat extrahiert. Die antibakterielle Wirksamkeit wurde gegen vier arzneimittelresistente Stämme von *Escherichia (E.) coli* und je fünf von *Klebsiella (K.) pneumoniae* und *Pseudomonas (P.) aeruginosa* geprüft, die aus dem Sputum von beatmungspflichtigen Patienten isoliert worden waren. Ermittelt wurde die antibakterielle Aktivität mittels Diskdiffusionsmethode. Für die beiden wirksamsten etherischen Öle wurde die minimale Hemmkonzentration (MHK) bestimmt.

**Ergebnisse:**

Die Ergebnisse des Antibiogramms zeigten, dass *Trachyspermum ammi* die größten Hemmzonen gegen alle Bakterien erzeugte, gefolgt von *Oliveria decumbens*. *Trachyspermum ammi* zeigte die höchste antibakterielle Aktivität gegen *E. coli*, während *Oliveria* *decumbens* am effektivsten gegen *K. pneumoniae* war. Bei den MHK-Tests wies Oliveria decumbens bei niedrigeren Konzentrationen eine stärkere antibakterielle Wirkung im Vergleich zu *Trachyspermum ammi* auf.

**Fazit:**

Erstmals wird über die antibakterielle Wirksamkeit der etherischen Öle aus vier Pflanzen, insbesondere *Trachyspermum ammi* und *Oliveria decumbens*, gegen multiresistente Bakterien, die von beatmungspflichtigen Patienten isoliert worden waren, berichtet. Beide Pflanzen zeigen ein vielversprechendes Potenzial als antibakterielle Mittel gegen die geprüpften Bakterien.

## Introduction

Antibacterial resistance has rapidly emerged as a pressing global health issue, placing a significant strain on healthcare systems. The scarcity of effective antibiotics has complicated the treatment of infections, making medical interventions and invasive procedures considerably riskier [1], [2]. According to the US Center for Disease Control and Prevention, over 2.8 million cases of antibiotic-resistant infections occur annually, leading to more than 35,000 deaths. Projections indicate that by 2050, deaths attributed to microbial resistance could rise to 10 million [3], [4], [5].

Antibiotic resistance is an adaptive response to antibacterial agents, stemming from the overuse and misuse of antibiotics. This has resulted in the emergence of new strains that differ from their predecessors, contributing to numerous health challenges [6]. The six primary pathogens associated with antibiotic-resistant fatalities are *Escherichia (E.) coli* followed by *Staphylococcus (S.) aureus, Klebsiella (K.) pneumoniae*, *Streptococcus pneumoniae, Acinetobacter baumannii*, and *Pseudomonas (P.) aeruginosa* [7], [8]. A key health priority is the development of effective antibacterial drugs to combat multidrug-resistant bacteria, particularly among Gram-negative pathogens [9].

In the past decade, significant strides have been made in exploring natural plant products as potential new antibacterial agents [10]. Natural products serve as valuable reservoirs of antibacterial compounds with considerable promise for addressing emerging bacterial strains [11]. Notably, the diversity and accessibility of natural plant compounds, along with their various mechanisms of action and established clinical efficacy, can be attributed to the presence of phenolic compounds (including simple phenols, phenolic acids, quinones, flavonoids, tannins, and coumarins), terpenoids, alkaloids, lectins, and polypeptides, which are fundamental to the antibacterial properties of medicinal plants [12], [13]. The varied climatic conditions in Iran have fostered a rich diversity of vascular-plant flora, making the utilization of this vast resource particularly significant in this context [14].

*Oliveria decumbens* [Apiaceae] is a herbaceous plant native to Iran, predominantly found in the southern and western regions. In traditional Iranian medicine, it is employed as a liver and heart tonic, as well as for its anti-diarrheal, antipyretic, and digestive properties. Furthermore, its antibacterial, antioxidant, antitumor, and insecticidal activities have been substantiated [15], [16].

*Zataria (Z.) multiflora* [Lamiaceae] thrives in southern and central Iran and is traditionally used for both culinary and medicinal purposes. The plant exhibits various pharmacological effects, including bronchodilation, vasodilation, and anti-inflammatory properties. The essential oil extracted from *Z. multiflora* demonstrates strong antibacterial activity against *E. coli, S. aureus*, and *Salmonella typhimurium* [16].

*Trachyspermum ammi* [Apiaceae] is extensively cultivated across Iraq, Iran, Afghanistan, Pakistan, and India. The essential oil derived from this plant is utilized in a wide array of medicinal applications, such as antibacterial, antifungal, anti-inflammatory, antioxidant, cytotoxic, antilithic, nematicidal, anthelmintic, and antifilarial treatments. Its seeds possess notable digestive and antiseptic properties and are primarily employed in traditional medicine to address intestinal disorders such as indigestion, flatulence, and diarrhea [17].

*Cuminum cyminum* [Apiaceae] is predominantly grown in arid and semi-arid regions, including Iran, China, Egypt, Saudi Arabia, as well as India. Traditionally, this plant is widely used in medicine for treating digestive issues, inflammatory conditions, nervous disorders, and toothache. In traditional Iranian medicine, *Cuminum cyminum* is utilized to treat conditions such as colic, diarrhea, indigestion, and flatulence, as well as to promote breast milk production. *Cuminum cyminum* also shows promise in inhibiting biofilm formation and possesses notable antibacterial properties against various bacterial pathogens, particularly gram-negative strains [18].

While numerous studies have explored the effects of these medicinal plants on different bacteria, there has been limited research specifically investigating the antibacterial properties of essential oils derived from medicinal plants against drug-resistant bacteria isolated from clinical samples. Consequently, the aim of this study was to examine the antibacterial effects of essential oils from several species of medicinal plants native to Iran on drug-resistant clinical microorganisms isolated from tracheal culture samples of patients on ventilators with hospital-acquired infections.

## Materials and methods

This study was carried out in April and May 2022 at the Cell and Molecular Research Center of Qom University of Medical Sciences, with support from the Qom University of Medical Sciences, under the ethics approval code IR.MUQ.REC.1401.068.

### Plant collection

The aerial parts of Oliveria decumbens were collected from Kazeroon city, Fars Province, at an altitude of 860 m. The aerial parts of *Zataria multiflora*, seeds of *Trachyspermum ammi*, and aerial parts of *Cuminum cyminum* were provided by a co-author, a pharmacognosist, from the Pharmaceutical Sciences Research Center, Shahid Beheshti University of Medical Sciences. All plant materials were authenticated by a pharmacognosist for verification.

### Essential oil preparation

The collected plant materials were dried in the shade. An accurate weight of 100 g was taken from each sample, which was then ground using an electric mill. Each 100-g portion of plant powder was distilled separately for three hours using a Clevenger apparatus. The extracted essential oils were collected from the Clevenger apparatus and stored in dark vials at 4°C in a refrigerator until required for experimentation.

### Sampling

13 clinical strains were obtained from the microbiology department of Shahid Beheshti University of Medical Sciences in Tehran, Iran. These included four strains of *E. coli*, five strains of *K. pneumoniae*, and four strains of *P. aeruginosa*. The strains were isolated from tracheal samples of patients in ICU departments across Tehran hospitals who were suffering from nosocomial infections. Notably, these strains exhibited high resistance to antibiotics, including the production of extended spectrum beta-lactamase (ESBL) and metallo beta-lactamase (MBL); both *P. aeruginosa* and *K. pneumoniae* showed resistance to all tested antibiotics [19]. Each strain was assigned a unique identification code. 

### Determining the antimicrobial properties of essential oils using the disk-diffusion method

Initially, bacteria were cultured on Brain Heart Infusion (BHI) agar (Ibresco, Iran) for 24 hours. A fresh bacterial suspension was then prepared to a concentration of 0.5 McFarland (1.5×10^8^ cfu/ml). Using a sterile swab, the suspension was evenly spread across Mueller Hinton Agar (MHA) (Ibresco, Iran) to create a lawn culture. Subsequently, 20 µl of each essential oil was placed on blank disks, and four disks containing each essential oil were positioned on the agar plate at regular intervals. After incubating for 24 hours at 37°C, the diameter of the inhibition zone was measured. The two essential oils that exhibited the most significant antibacterial effect were selected for further testing.

### Determining the antibacterial properties of essential oils using the minimum inhibitory concentration (MIC) method

The MIC of the two essential oils with the largest inhibition zones was assessed. To achieve this, an eight-fold dilution series (50%, 25%, 12.5%, 6.25%, 3.125%, 1.56%, 0.78%, and 0.39% v/v) of the essential oils was prepared in a 96-well microplate (SPL, South Korea) and added to the wells. Bacteria, prepared to half McFarland’s standard, were then introduced into each well. Additionally, one well containing only the culture medium and bacteria (without essential oil) served as a positive control, while another well containing essential oil and culture medium (without bacteria) acted as a negative control. After incubating for 24 hours at 37°C, turbidity was assessed, and the last well showing no turbidity (indicating no bacterial growth) was recorded as the MIC. 

## Results

The antibiotic resistance profile of *K. pneumoniae, P. aeruginosa*, and *E. coli* isolates revealed a high level of resistance to multiple antibiotics, emphasizing the need for alternative antibacterial agents. The detailed resistance patterns for each bacterial strain are outlined in Table 1 (Tab. 1). 

The antibiogram analysis revealed that among the tested essential oils, *Trachyspermum ammi* exhibited the highest antibacterial activity against all bacterial strains, followed closely by Oliveria decumbens. Specifically, *Trachyspermum ammi* demonstrated the most potent inhibitory effect against *E. coli*, whereas *Oliveria decumbens* was most effective against *K. pneumoniae*. The detailed inhibition zone diameters for each bacterial strain are presented in Table 2 (Tab. 2). 

According to the MIC results, although both extracts had inhibitory effects equal to 0.39 v/v, a greater number of strains were inhibited by *Oliveria decumbens* extract at this concentration, and therefore it showed a better antibacterial effect than *Trachyspermum ammi* extract. The complete MIC data are presented in Table 3 (Tab. 3).

## Discussion

Antibacterial resistance poses a significant threat to global health, leading to substantial challenges within healthcare systems [20], [21], [22]. Among the antibiotic-resistant pathogens are *E. coli, K. pneumoniae*, and *P. aeruginosa*, which are responsible for numerous fatalities, particularly among hospitalized patients [7]. *E. coli* is one of the most prevalent causes of infections globally. The widespread use of cephalosporins and fluoroquinolones has led to a dramatic increase in multidrug-resistant (MDR) strains. These findings underscore the urgent need for alternative treatments for MDR *E. coli* infections [23], [24], [25].

*P. aeruginosa* is an opportunistic bacterial pathogen linked to various infections, including nosocomial infections, endocarditis, pneumonia, urinary tract infections, septicemia, as well as skin, eye, and ear infections [26]. Similarly, *K. pneumoniae* is a significant Gram-negative opportunistic pathogen associated with a range of infectious diseases, such as urinary tract infections, bacteremia, pneumonia, and liver abscesses. The emergence of MDR strains of *K. pneumoniae* and their rapid global spread is particularly concerning [27]. In light of widespread antibiotic resistance, researchers are actively seeking more effective compounds as alternatives. Recently, there has been growing interest in using plants for infection treatment [28]. Therefore, this study evaluates three plant types that have been previously researched against clinical strains with high antibiotic resistance.

In our research, the essential oil of the Trachyspermum ammi plant exhibited the strongest antibacterial effect across all tested bacteria, especially against *E. coli*. Jebelli Javan et al. [29] identified the synergistic effects of *Trachyspermum ammi* essential oil combined with propolis ethanolic extract on foodborne bacteria. Another study involving Modareskia [30] found that *Trachyspermum ammi* essential oil demonstrated bacteriostatic activity against *S. aureus* and *E. coli*. Additionally, it was shown that the essential oil of *Trachyspermum ammi*, rich in monoterpenes, possesses considerable antibacterial properties against MDR *S. aureus* and *P. aeruginosa* [31].

Following *Trachyspermum ammi, Oliveria decumbens* exhibited the next highest antibacterial effect in our study. Mahboubi et al. [32] found that Gram-positive bacteria were generally more sensitive than Gram-negative strains using standard microbial strains, with P. aeruginosa displaying notable resistance. However, the advantage of our study is that it focused on antibiotic-resistant clinical strains. Eftekhari et al. [33] also explored the antibacterial properties of *Oliveria decumbens* essential oil using the disk diffusion method on both Gram-negative (*E. coli, P. aeruginosa*) and Gram-positive bacteria (*S. epidermidis, S. aureus*). Their findings indicated strong antibacterial activity against *S. aureus, S. epidermidis*, and *E. coli*, but no effect against *P. aeruginosa* at concentrations up to 20.4 µg/mL. In our study, the essential oil of *Zataria multiflora* demonstrated moderate antibacterial activity. Research conducted by Golkar et al. [34] evaluated the antibacterial effects of *Zataria multiflora* on *E. coli* (ATCC35218), reporting a non-growth halo diameter of 6.4 mm and a minimum inhibitory concentration (MIC) of 5 µg/mL. In contrast, our findings indicated an average non-growth halo diameter of 19 mm for *E. coli*. A review by Khaledi et al. [35] generally confirmed the antibacterial properties of thyme against *P. aeruginosa* under laboratory conditions. Interestingly, our previous study highlighted the superior efficacy of *Zataria multiflora* against clinical strains of *P. aeruginosa* [36].

Additionally, another study assessed the antibacterial properties of essential oils from *Artemisia kermanensis*, *Lavandula officinalis*, and *Zataria multiflora* against *S. aureus* (ATCC 25923), *P. aeruginosa* (PTCC 1310), and *K. pneumoniae* (PTCC 1053). The results indicated that all three essential oils exhibited inhibitory effects, with *Zataria multiflora* demonstrating the most significant antibacterial activity against the tested strains [37].

In the present investigation, *Cuminum cyminum* essential oil exhibited the weakest antibacterial properties. On average, it had the least impact on *E. coli* compared to other bacteria. A study by Oroojalian et al. [38] explored the effects of *Carum copticum, Bunium persicum, *and *Cuminum cyminum* on various bacteria, including *S. aureus, B. cereus*,* Listeria monocytogenes*, *E. coli* O157:H7, and *Salmonella enteritidis*. They found that while the MIC values for the essential oils of *Bunium persicum* and *Cuminum cyminum* were less effective than that of *Carum copticum*, their combined use showed promising results, particularly against Gram-positive bacteria.

## Limitations

The study had several limitations that may affect the interpretation and generalizability of its findings. Firstly, the essential oils were extracted using only the Clevenger apparatus, potentially overlooking variations in antibacterial properties that might arise from different extraction methods. Furthermore, the in-vitro nature of the study does not account for the complexities of human infections, such as biofilm formation and host immune responses. Additionally, while the study identified *Trachyspermum ammi* and *Oliveria decumbens* as effective antibacterial agents, it did not explore the specific mechanisms of action or assess potential cytotoxicity, which are crucial for evaluating their safety and therapeutic potential in clinical settings. Lastly, one of the limitations of the study was the completion of the *Trachyspermum ammi* essential oil and the failure to calculate the MIC value for the *E. coli*. Limited availability of sufficient plant material for extraction resulted in incomplete testing of *Trachyspermum ammi* essential oil, preventing a comprehensive evaluation of its antibacterial efficacy.

## Conclusion

This study highlights the promising potential of essential oils from medicinal plants as effective antibacterial agents against antibiotic-resistant bacteria isolated from ventilator-dependent patients. Given the growing global concern surrounding antibacterial resistance, our findings underscore the importance of exploring natural alternatives to conventional antibiotics.

The essential oils extracted from *Trachyspermum ammi* and *Oliveria decumbens* demonstrated significant antibacterial activity, with *Trachyspermum ammi* exhibiting the largest zones of inhibition across all tested strains, particularly against *E. coli*. Conversely, *Oliveria decumbens* showed remarkable efficacy against *K. pneumoniae*, especially at lower concentrations during the MIC assessments. These results suggest that the essential oils from these plants may serve as valuable adjuncts in the treatment of infections caused by drug-resistant pathogens, potentially offering new avenues for therapeutic strategies in clinical settings.

## Notes

### Authors’ ORCIDs

- Khahan-Yazdi I: 0000-0002-6034-955X
- Mohammadamin Shabani: 0000-0002-2333-8122
- Tajvidi-Monfared M: 0000-0003-3179-2189
- Taheri Kharameh Z: 0000-0002-9968-7951
- Mojab F: 0000-0003-2415-2175
- Shams S: 0000-0002-3701-3126
- Vahidi Emami H: 0000-0002-5355-6921

### Funding

This study was funded by Qom University of Medical Sciences and Health Services.

### Acknowledgments

This research represents the culmination of a project carried out at Qom University of Medical Sciences, under the Code of IR.MUQ.REC.1401.068. The project received support from both Qom University of Medical Sciences and the National Elite Foundation. Consequently, the researchers would like to extend their heartfelt gratitude and appreciation to all individuals who contributed to this study.

### Competing interests

The authors declare that they have no competing interests.

## Figures and Tables

**Table 1:**
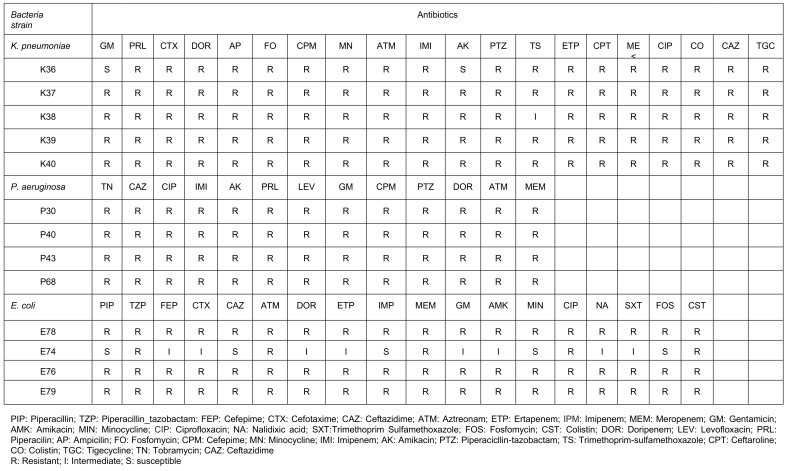
Resistance of bacterial strains of *K. pneumoniae*, *P. aeruginosa* and *E. coli* against different antibiotics

**Table 2:**
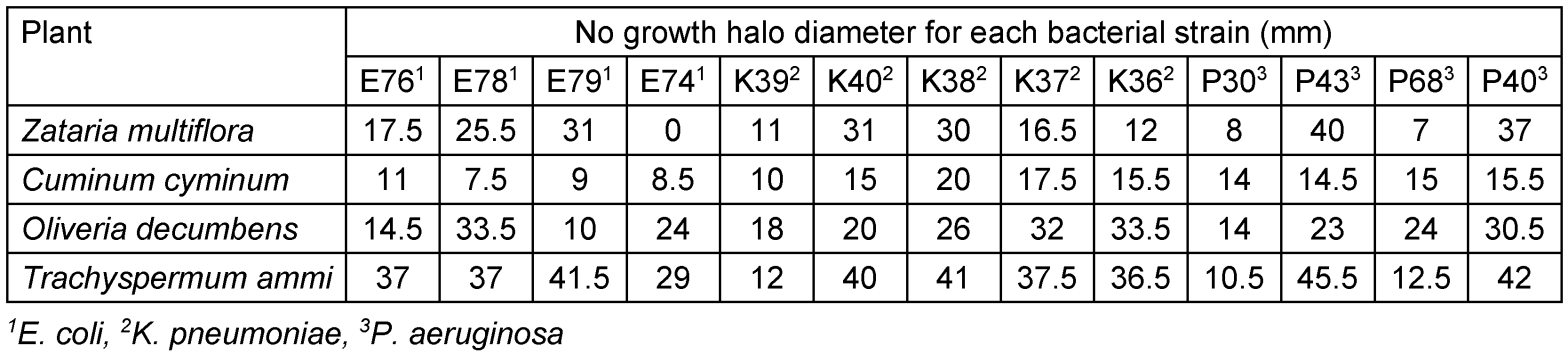
Antibacterial effect of essential oils of *Zataria multiflora*, *Cuminum cyminum*, *Oliveria decumbens* and *Trachyspermum ammi* by disk diffusion method

**Table 3:**
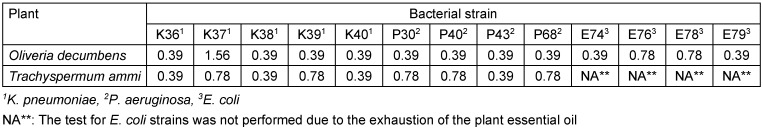
Antibacterial effect of the essential oils of *Oliveria decumbens* and *Trachyspermum ammi *by MIC method (v/v)
